# Noninvasive Ventilation Weaning in Acute Hypercapnic Respiratory Failure due to COPD Exacerbation: A Real-Life Observational Study

**DOI:** 10.1155/2019/3478968

**Published:** 2019-03-25

**Authors:** Paola Faverio, Anna Stainer, Federica De Giacomi, Grazia Messinesi, Valentina Paolini, Anna Monzani, Paolo Sioli, Irdi Memaj, Oriol Sibila, Paolo Mazzola, Alberto Pesci

**Affiliations:** ^1^School of Medicine and Surgery, University of Milan Bicocca, Respiratory Unit, San Gerardo Hospital, ASST Monza, Monza, Italy; ^2^Respiratory Department, Hospital de La Santa Creu I Sant Pau, Autonomous University of Barcelona and Biomedical Research Institute Sant Pau (IIB Sant Pau), Barcelona, Spain; ^3^School of Medicine and Surgery, University of Milan Bicocca, Acute Geriatrics Unit, San Gerardo Hospital, ASST Monza, Monza, Italy

## Abstract

The most recent British Thoracic Society/Intensive Care Society (BTS/ICS) guidelines on the use of noninvasive ventilation (NIV) in acute hypercapnic respiratory failure (AHRF) suggest to maximize NIV use in the first 24 hours and to perform a slow tapering. However, a limited number of studies evaluated the phase of NIV weaning. The aim of this study is to describe the NIV weaning protocol used in AHRF due to acute exacerbation of chronic obstructive pulmonary disease (AE-COPD), patients' characteristics, clinical course, and outcomes in a real-life intermediate respiratory care unit (IRCU) setting. We performed a retrospective study on adult patients hospitalized at the IRCU of San Gerardo Hospital, Monza, Italy, from January 2015 to April 2017 with a diagnosis of AHRF due to COPD exacerbation. The NIV weaning protocol used in our institution consists of the interruption of one of the three daily NIV sessions at the time, starting from the morning session and finishing with the night session. The 51 patients who started weaning were divided into three groups: 20 (39%) patients (median age 80 yrs, 65% males) who completed the protocol and were discharged home without NIV (Completed Group), 20 (39%) did not complete it because they were adapted to domiciliary ventilation (Chronic NIV Group), and 11 (22%) interrupted weaning *ex abrupto* mainly due to NIV intolerance (Failed Group). Completed Group patients were older, had a higher burden of comorbidities, but a lower severity of COPD compared to Chronic NIV Group. Failed Group patients experienced higher frequency of delirium after NIV discontinuation. None of the patients who completed weaning had AHRF relapse during hospitalization. While other NIV weaning methods have been previously described, our study is the first to describe a protocol that implies the interruption of a ventilation session at the time. The application of a weaning protocol may prevent AHRF relapse in the early stages of NIV interruption and in elderly frail patients.

## 1. Introduction

Bilevel noninvasive ventilation (NIV) is a milestone in the treatment of acute hypercapnic respiratory failure (AHRF) in patients with acute exacerbation of chronic obstructive pulmonary disease (AE-COPD) [1, 2]. Unfortunately, only a limited number of studies evaluated the weaning from NIV once acidosis and respiratory distress are solved, proposing different weaning methods but not clearly establishing the superiority of one over the others [3–6]. A recently published randomized controlled trial (RCT) questioned the utility of the weaning phase [5]. In this RCT, Sellares et al. compared the prolongation of NIV for three nights after recovery from an AHRF episode to direct NIV discontinuation in COPD patients without previous domiciliary ventilation [5]. No differences were observed in regard to prevention of AHRF relapse, hospital readmission, or mortality [5]. Furthermore, the application of a weaning phase resulted in a prolonged intermediate respiratory care unit (IRCU) stay. These results seem to suggest that NIV can be directly discontinued once the acute episode is solved. However, as in all RCTs, very elderly patients, not fully cooperative and with several comorbidities, have rarely been included, although they often represent a consistent part of the COPD population with AHRF admitted to IRCUs [5]. In such a frail group, many factors may play a role in AHRF relapse after NIV discontinuation, including other respiratory comorbidities, delirium, and sarcopenia [7]. Therefore, the actual need of weaning from NIV in a real-life cohort of patients with AHRF and AE-COPD and the best way to perform it still need to be determined.

Our aim is to present and test the feasibility of the NIV weaning strategy used in patients with AHRF due to AE-COPD admitted in our IRCU. Furthermore, we describe the characteristics, clinical course, and outcomes of the cohort of patients who underwent the NIV weaning protocol.

## 2. Materials and Methods

### 2.1. Study Design

This was a retrospective cohort study on adult patients hospitalized in the IRCU of San Gerardo Hospital, Monza, Italy, from January 2015 to April 2017 with a diagnosis of AE-COPD. The study was approved by the local Institutional Review Board (#684-May 2017) and did not require patients' informed consent due to the retrospective design.

The following data were retrospectively collected from patients' charts: demographics, comorbidities and Charlson comorbidity index [8], COPD characteristics (including pulmonary function tests performed at the previous available outpatient visit during clinical stability, number of exacerbations and hospitalizations during the prior year, and COPD phenotype—emphysema, chronic bronchitis, asthma-COPD overlap, and bronchiectasis-COPD overlap [9, 10]), severity scores at admission (acute physiology and chronic health disease classification system II (APACHE II) [11] and sequential organ failure assessment (SOFA) [12]), radiological imaging during hospitalization (either chest X-ray, computerized tomography scan, or both), arterial blood gases (ABG), days of NIV application and hospital length of stay, presence of delirium during hospitalization, and NIV parameters. Follow-up data, including three-month mortality and hospitalization rate, were also retrospectively collected from outpatient clinic charts. All patients discharged from our IRCU with a diagnosis of AE-COPD were followed-up after 3 months from discharge at our COPD outpatient clinic; those who did not present at the visit received a phone call to reprogram it.

### 2.2. AE-COPD Definition

AE-COPD was defined as acute worsening of respiratory symptoms that resulted in additional therapy, including antibiotics, systemic corticosteroids, and increased bronchodilator therapy [13]. In our study population, AE-COPD was the primary admission diagnosis according to the physician in charge. Patients who also had pneumonia or congestive heart failure as secondary diagnoses were not excluded from the study, and these variables were reported in the Results section.

### 2.3. NIV Initiation Criteria and Indication to Chronic Domiciliary NIV

In accordance with the protocol adopted by our institution, bilevel NIV was initiated in patients with AHRF (arterial carbon dioxide partial pressure (PaCO_2_) > 45 mmHg) due to AE-COPD in case of respiratory acidosis (pH ≤ 7.35) and/or respiratory distress. Respiratory distress was defined as the respiratory rate (RR) > 25 associated with use of respiratory accessory muscles. In case of pH < 7.25, decision regarding NIV initiation was shared with the intensive care unit (ICU) team.

All patients were ventilated with pressure support ventilation modality. The ventilators available in our IRCU were Respironics Esprit and Resmed Elisée 150. Full face and oronasal interfaces were used.

In our institution, indications for domiciliary NIV in COPD patients were persistent daytime hypercapnia after at least two episodes of AHRF requiring NIV in the prior year; and comorbidities such as obstructive sleep apnea or obesity-hypoventilation syndrome, in accordance with guidelines for each disease [1, 14, 15].

### 2.4. NIV Disconnection and NIV Weaning Initiation Criteria

NIV disconnection was attempted when all the following were concurrently met:

- Adequate oxygenation defined as arterial oxygen partial pressure to fraction of inspired oxygen ratio (PaO_2_/FiO_2_) > 200 mmHg during NIV with fraction of inspired oxygen (FiO_2_) < 0.5
- pH > 7.35, RR < 25 without use of respiratory accessory muscles
- Hemodynamic stability (assessed through heart rate and blood pressure)
- Kelly score [16] ≤ 2

The weaning phase began when all the following criteria were reached after at least 1 hour of disconnection from NIV and by administering oxygen through a venturi mask with a FiO_2_ titrated in order to maintain peripheral oxygen saturation (SpO_2_) 88–92%:

- pH > 7.35, RR < 25 without use of respiratory accessory muscles
- Hemodynamic stability
- Kelly score [16] ≤ 2

### 2.5. NIV Weaning Protocol

**Figure 1:**
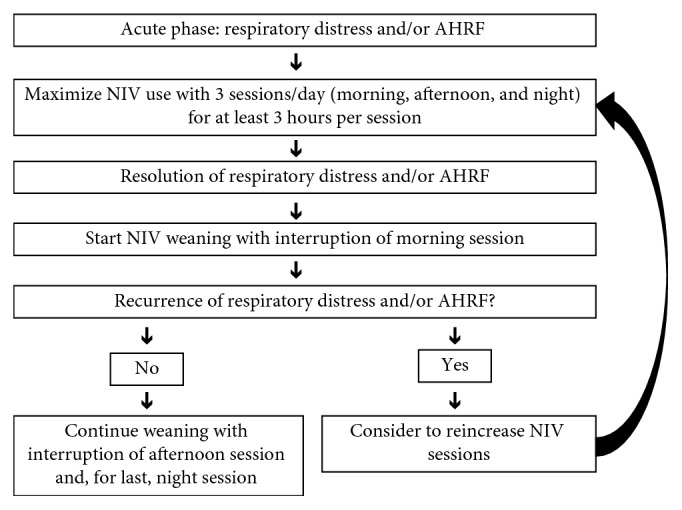
NIV weaning protocol. AHRF, acute hypercapnic respiratory failure; NIV, noninvasive ventilation.

The weaning protocol used in our institution is summarized in Figure 1. The term “NIV session” was used to indicate the continuous application of NIV for at least 3 hours to the maximum duration tolerated by the patient, with interruption only for oral therapy administration and/or feeding. During the acute initial phase, the treatment is maximized with 3 NIV sessions per day (morning, afternoon, and night) (Figure 1). In our NIV weaning protocol, the patient starts NIV weaning with interruption of the morning session. If there is no recurrence of respiratory distress or AHRF, the patient continues weaning with interruption of the afternoon session and, for last, of the nocturnal session. The NIV sessions were interrupted one per day (e.g., day 1: interruption of the morning session; day 2: interruption of the morning and afternoon session; day 3: interruption of all sessions—morning, afternoon, and night). The number of days at each stage of weaning (NIV during afternoon and night or nocturnal NIV only) was at physician discretion. During weaning, NIV pressures, both inspiratory positive airway pressure (IPAP) and expiratory positive airway pressure (EPAP), were maintained on the same personalized settings used to reverse AHRF.

During NIV weaning, every patient underwent pulmonary rehabilitation between NIV sessions with positive expiratory pressure (PEP) and incentive spirometry (IS) devices. In patients with residual mucus encumbrance, we used PEP devices (i.e., PEP bottle or TheraPEP, Smiths Medical) set at low pressure (8 cm H_2_O) asking the patient to blow as long as possible repeatedly without achieving fatigue, in order to improve airway clearance. In patients with poor thoracoabdominal movements, we used IS devices in association with stretching exercises aimed at preventing atelectasis or local hypoventilation. The patient was invited to repeatedly inspire slowly through the device (Coach II, Smiths Medical) lifting a piston up to the limit set at 70–80% of inspiratory capacity and then hold breath for five seconds. Pulmonary rehabilitation was set and carried out by a respiratory therapist up to two times per day; additional sessions were kept by nurses.

NIV weaning failure occurred if any of the following appeared during the execution of the NIV weaning protocol:

- pH ≤ 7.35 and/or respiratory distress
- Hemodynamic instability
- Kelly score [16] > 2

### 2.6. Delirium Definition and Criteria for Identification

The chart-based method (CBM) was used to retrospectively identify delirium on patients' charts [17, 18], searching for key terms for identification of delirium (inattention, disorientation, hallucinations, agitation, and inappropriate behavior) and for any evidence of acute mental status change.

We temporally divided the presence of delirium into two subgroups: delirium during NIV and delirium after NIV discontinuation, in order to evaluate the possible role of this complication in association with NIV weaning and clinical outcomes.

### 2.7. Outcomes

The following outcomes were considered for all patients who initiated the weaning phase:

- Primary outcomes: new worsening of gas exchange (pH ≤ 7.35, RR > 25, and/or respiratory distress) after completion of weaning protocol and in-hospital mortality
- Secondary outcomes: three-month mortality and three-month hospitalization rate

### 2.8. Statistical Analysis

Data were analyzed using SPSS 21.0 for MAC OS (SPSS Inc., Chicago, IL, USA). Baseline characteristics of the study population, ABGs, ventilation parameters, and outcomes, were considered for statistical analysis. We assessed normality distribution for continuous variables with the Kolmogorov–Smirnov test. Continuous variables were expressed as median (interquartile range (IQR): 25th–75th percentile) and compared using the Kruskal–Wallis nonparametric ANOVA test. Categorical data were expressed as frequencies and percentages and compared using the chi-square test. All tests were 2-tailed, and a *P* value < 0.05 was considered statistically significant.

## 3. Results

### 3.1. Characteristics of Study Population

**Figure 2:**
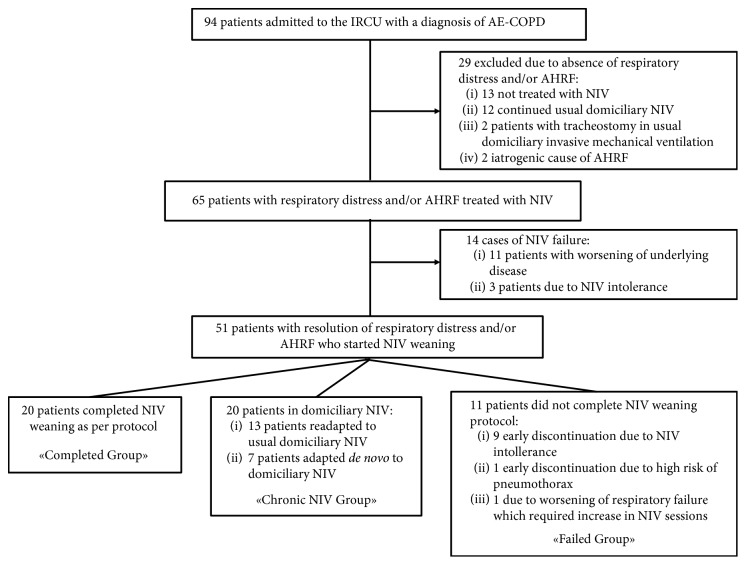
Flow chart of study population. IRCU, intermediate respiratory care unit; COPD, chronic obstructive pulmonary disease; AHRF, acute hypercapnic respiratory failure; NIV, noninvasive ventilation.

Among the 94 patients hospitalized in the IRCU with a diagnosis of COPD exacerbation during the study period, we focused on 51 patients (median age 79 yrs, 53% males) who were started on NIV weaning after an episode of AHRF (Figure 2).

Of these 51 patients, 20 (median age 80 yrs, 65% males) completed the protocol (Completed Group), 20 (median age 65 yrs, 35% males) did not complete the weaning because they were adapted to chronic domiciliary ventilation (Chronic NIV Group), and 11 (median age 82 yrs, 65% males) did not complete the protocol for other clinical conditions (Failed Group) (Figure 2).

Clinical conditions for which Failed Group did not complete weaning included NIV intolerance, high risk of pneumothorax due to severe bullous emphysema, and NIV weaning failure, as defined in Section 2, which required an increase in NIV sessions (Figure 2). None of the 51 patients developed facial skin lesion; however, three patients developed gastric distension associated with nausea in one case.

Demographics, comorbidities, and COPD characteristics of the three study groups are summarized in Table 1. Completed Group patients were older and had a higher burden of comorbidities, evaluated through the Charlson index. In regard to specific comorbidities, Completed Group presented the highest frequency of cardiovascular diseases, while Chronic NIV Group showed more frequently obesity-hypoventilation syndrome and mood disorders. Patients in Completed Group presented more frequently a chronic bronchitis phenotype and had a lower number of COPD exacerbations and hospitalizations in the prior year. However, this finding is related to the definition itself of patients in “Chronic NIV Group” that had to present at least two episodes of AHRF, requiring NIV in the prior year to receive chronic nocturnal domiciliary NIV. Only two patients in Completed Group received a do-not-resuscitate/do-not-intubate (DNR/DNI) order, which was significantly less frequent than in Failed Group.

There was no between-group difference in the prevalence of delirium during NIV and during hospitalization as a whole (Table 1). The prevalence of delirium after NIV discontinuation, however, was greater in the Failed Group than in the Completed Group.

### 3.2. Severity on Admission and Arterial Blood Gases Evaluation

**Figure 3:**
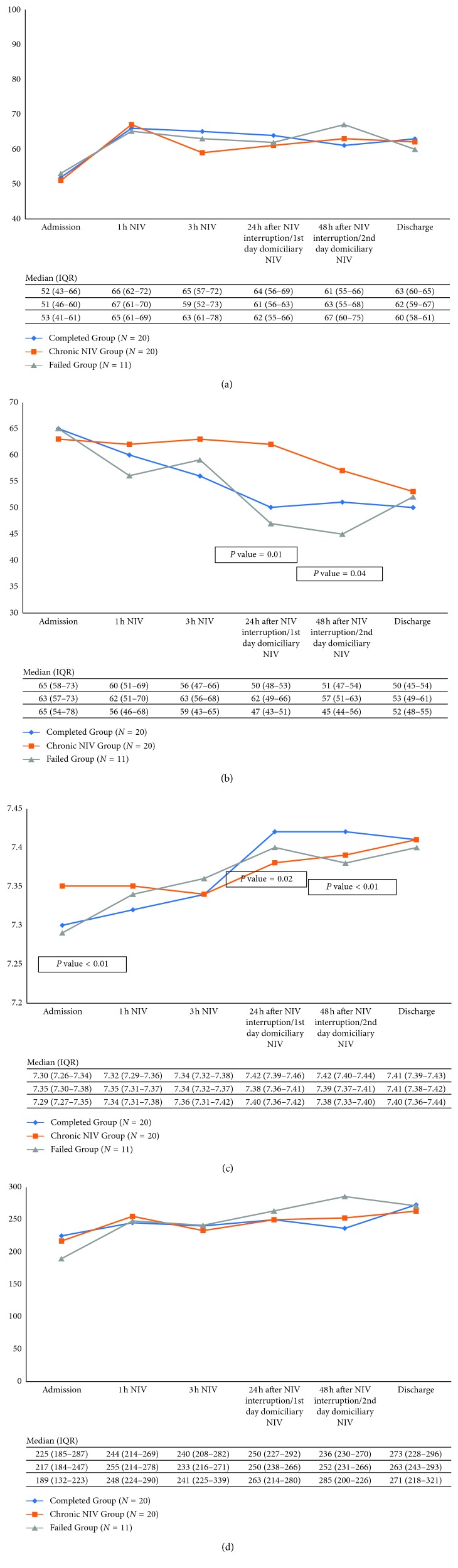
Arterial blood gases at different stages both during NIV and after NIV discontinuation. ABG, arterial blood gas; IQR, interquartile range 25th–75th percentile; PaO_2_, arterial oxygen partial pressure (mmHg); PaCO_2_, arterial carbon dioxide partial pressure (mmHg); P/F = PaO2/FiO_2_, arterial oxygen partial pressure to fraction of inspired oxygen ratio; NIV, noninvasive ventilation. (a) ABG: pO_2_, (b) ABG: pH, (c) ABG: pCO_2_, and (d) ABG: P/F.

APACHE II score was greater in the Failed Group compared to the other two study groups. In contrast, there was no between-group difference in the SOFA score (Table 2). Also, pH values of the ABG performed at admission were lower in Completed Group and Failed Group in comparison with Chronic NIV Group (7.30, 7.29, and 7.35, respectively; *P* value <0.01) (Figure 3). There were no between-group differences in concomitant causes of AHRF (Table 2).

Furthermore, we analyzed the ABGs performed at different stages both during and after NIV discontinuation (Figure 3). No difference was observed between groups with the exception of lower PaCO_2_ values in Completed Group and Failed Group compared to Chronic NIV Group at ABGs performed at 24 and 48 h after NIV discontinuation, but not at the ABG performed at discharge.

### 3.3. NIV Duration and Parameters

Compared to the Completed Group, the duration of NIV was shorter in the Failed Group and longer in the Chronic NIV Group (for the latter group, days on nocturnal domiciliary NIV were included in the computation of total NIV duration, because it was artificial to exactly locate the moment when weaning was finished) (Table 3). However, no differences were found between groups in regard to number of days with maximized NIV before weaning (3 sessions per day) with a median of one day for each group and length of hospital stay.

For patients in Completed Group and Failed Group, lower NIV pressures (both IPAP and EPAP) were used in comparison with Chronic NIV Group. In this latter group, NIV pressures used during the acute phase were similar to those titrated for the chronic domiciliary NIV use, as shown in Table 3.

### 3.4. Outcomes

Three patients, all in Failed Group, experienced relapse of AHRF during the first 48 hours from NIV discontinuation (*P* < 0.01), and two of them died during the hospitalization (both of them had received a DNR/DNI order due to the severity of comorbidities) (Table 4). No difference was observed between groups in regard to secondary outcomes.

## 4. Discussion

While other NIV weaning methods have been previously described [3–6], our study is the first to describe a protocol that implies the interruption of a ventilation session at the time. None of the patients who completed weaning experienced AHRF relapse or died in the course of hospitalization. A considerable proportion of patients, particularly those younger, with a lower burden of comorbidities, but a higher severity of COPD, was initiated on chronic domiciliary NIV. Delirium should be taken into account among the factors that can complicate the clinical course of ventilated patients with AHRF due to AE-COPD even in the weaning phase.

Despite the wide range of studies available on weaning from invasive mechanical ventilation [19], few studies have investigated the strategies to wean patients from NIV [3–6]. Similarly to Damas et al., who described a weaning protocol that maintains the same number of sessions per day but provides a progressive reduction of hours for each NIV session, we did not observe recurrence of AHRF during hospitalization after weaning [4]. Two recently published RCTs by Sellares et al. and Lun et al. comparing immediate *vs*. stepwise NIV withdrawal did not show any benefit from the use of a weaning strategy in preventing relapse of AHRF after NIV discontinuation, suggesting that NIV can be directly discontinued in COPD patients once the episode of AHRF is solved [5, 6]. However, when looking at the weaning phase itself, in our population, only one patient failed the first weaning attempt because of worsening respiratory failure. In the studies by Sellares et al. and Lun et al., 19% and 44% of patients, respectively, in the direct discontinuation groups failed the first withdrawal attempt [5, 6]. These percentages were higher than in the stepwise withdrawal (5% and 26%, respectively) [5, 6]. In spite of this variable not being included among study outcomes, it is important to consider that the application of a weaning protocol may prevent respiratory function deterioration even in the early stages of NIV interruption.

Furthermore, both the aforementioned studies recruited only fully cooperative patients with a limited burden of comorbidities. In a real-life setting, it is often necessary to apply NIV to elderly patients with a high burden of comorbidities (median Charlson comorbidity index in our population ranged between 4 and 7), not fully cooperative (as demonstrated by the prevalence of delirium in our cohort) and who received a DNR/DNI order (16% of cases in our cohort). These frail patients often excluded from clinical trials could be the ones most benefiting from weaning protocols.

Only a minority of patients in our study completed the weaning protocol, while a considerable proportion (39%) was initiated on chronic domiciliary NIV. These patients were younger, with a lower burden of comorbidities but a higher severity of COPD compared to patients who completed weaning. These data suggest that patients who are most likely to receive chronic domiciliary NIV are those with a longer life expectancy and more severe COPD.

According to the CBM, 23% of patients in our cohort had a diagnosis of delirium. A meta-analysis published in 2012 reported a prevalence of delirium of 37% in patients on NIV, which was higher than the one generally presented in hospitalized nonventilated patients (10–31%) [20]. Furthermore, the risk of NIV failure in patients with delirium was 2.12 times higher than in those without delirium. Our study groups showed similar prevalence of delirium during NIV, whereas, after NIV discontinuation, patients who discontinued NIV *ex abrupto* showed a higher prevalence compared to those who completed the weaning protocol. We postulate that delirium, on a case-by-case basis, might be both a cause and a consequence of NIV intolerance and discontinuation. It is important to emphasize that patients who had a sudden NIV interruption were also the oldest and had the highest burden of comorbidities and the highest percentage of DNR/DNI orders. All these factors, together with the higher incidence of delirium after NIV discontinuation, may have played a role in the worse clinical outcomes (AHRF relapse after NIV discontinuation and in-hospital mortality) observed in this group of patients. However, as a potentially preventable and treatable condition, an early diagnosis is fundamental in frail patients, such as those ventilated for AHRF, not only in the acute phase but also in the weaning process.

Furthermore, although patients in the “Failed group” interrupted NIV earlier compared to other groups, no differences were observed in regard to the length of hospital stay, once again indicating that these patients had a complicated clinical condition that required a prolongation of the hospitalization regardless of NIV use.

Limitations of the present study to be acknowledged mainly address the small sample size and the monocentric retrospective design, as well as the absence of a control group to compare different weaning methods. Delirium was assessed through a retrospective, although validated [18], method that may have under- or overestimated the actual incidence of this complication. Since our purpose was to picture the use of a NIV weaning method in a real-life scenario, we included in the study patients with AE-COPD as primary diagnosis and secondary diagnosis of pneumonia and congestive heart failure, as declared in Section 2. However, the study population was too small to allow analyses and speculations on the different application and utility of a NIV weaning protocol according to the concomitant causes of AHRF.

Future studies should address the question of the utility of NIV weaning and the best way to apply it. Assuming that the paradigm “one size fits all” is incorrect, it would be of paramount importance to determine which population might benefit the most from the application of NIV weaning protocols, including frail elderly patients.

## 5. Conclusions

In our cohort, the completion of a NIV weaning protocol based upon the interruption of one of the three daily NIV sessions at the time is associated with the absence of negative outcomes, including AHRF relapse and in-hospital mortality. Patients with AHRF should be carefully evaluated for presence of delirium both during and after NIV discontinuation.

## Figures and Tables

**Table 1:** Demographics, comorbidities, COPD characteristics, and presence of delirium in study population.

	Completed Group (*N*=20)	Chronic NIV Group (*N*=20)	Failed Group (*N*=11)	*P* value
Demographics, *n* (%)				
Age (yrs), median (IQR)	79 (74–85)	65 (55–75)	82 (77–84)	**<0.01**
Males	13 (65)	7 (35)	7 (64)	0.65
BMI (kg/m^2^), median (IQR)	24.9 (21.1–27.1)	26.2 (24.6–29.1)	23.9 (21–23.4)	0.12
Current or prior smokers	20 (100)	20 (100)	11 (100)	—
Do-not-resuscitate order	2 (10)	1 (5)	5 (45)	**0.02**
Comorbidities, *n* (%)				
Charlson comorbidity index, median (IQR)	6.75 (6-7)	4 (3–5)	7 (6–8)	**<0.01**
Diabetes mellitus	4 (20)	2 (10)	2 (18)	0.09
Chronic heart diseases	12 (60)	4 (20)	4 (36)	0.09
Prior myocardial infarction	9 (45)	0	4 (36)	0.26
Cerebrovascular diseases	2 (10)	3 (15)	2 (18)	0.51
Chronic liver disease	2 (10)	1 (5)	0	0.25
Solid tumor	2 (10)	1 (5)	2 (18)	0.59
Hematologic malignancy	1 (5)	0	0	0.28
Obstructive sleep apnea syndrome	1 (5)	4 (20)	1 (9)	0.055
Kyphoscoliosis	0	0	2 (18)	0.03
Obesity-hypoventilation syndrome	0	4 (20)	0	0.63
Dementia	0	0	0	—
Psychiatric disorders	2 (10)	9 (45)	3 (27)	0.16
Chronic renal failure	2 (10)	0	2 (18)	0.63
COPD characteristics, *n* (%)				
FEV1 (L), median (IQR)	0.79 (0.62–0.99)	0.49 (0.36–0.82)	0.89 (0.69–1)	**0.03**
FEV1 (%), median (IQR)	42 (35–50)	33 (17–45)	40 (32–49)	0.21
GOLD 4^*∗*^	16 (80)	15 (75)	7 (64)	0.60
Emphysema phenotype	3 (15)	0	5 (45)	0.87
Chronic bronchitis phenotype	15 (75)	9 (45)	4 (36)	**0.03**
Asthma-COPD overlap phenotype	2 (10)	7 (35)	2 (18)	0.39
Bronchiectasis-COPD overlap phenotype	0	3 (15)	0	0.68
No. of exacerbations in the previous year, median (IQR)	1 (0-1)	3 (2–5)	1 (0–2)	0.07
No. of hospitalizations in the previous year, median (IQR)	1 (0-1)	2 (0–5)	1 (0–2)	**0.01**
Long-term oxygen therapy	10 (50)	17 (85)	5 (45)	0.81
Prior domiciliary NIV	0	17 (85)	0	0.27
Delirium, *n* (%)				
Delirium according to CBM	4 (20)	4 (20)	4 (36)	0.36
Delirium during NIV	4 (20)	4 (20)	3 (27)	0.68
Delirium after NIV discontinuation	0	—	3 (27)	—

BMI, body mass index; CBM, chart-based method; COPD, chronic obstructive pulmonary disease; FEV1, forced expiratory volume in the 1st second; GOLD, global initiative for chronic obstructive lung disease; IQR, interquartile range 25th–75th percentile; NIV, noninvasive ventilation. ^*∗*^Classification of airflow limitation severity based on postbronchodilator FEV1 [13].

**Table 2:** Severity scores on admission and concomitant causes of AHRF.

	Completed Group (*N*=20)	Chronic NIV Group (*N*=20)	Failed Group (*N*=11)	*P* value
Severity scores, median (IQR)				
Apache II	19 (17–21)	16 (15–19)	20 (14–21)	**0.02**
SOFA score	3 (2–4)	3 (2-3)	3 (2-3)	0.80
Concomitant causes of AHRF, *n* (%)				
Pneumonia	5 (25)	5 (25)	4 (36)	0.55
Congestive heart failure	14 (70)	8 (40)	7 (64)	0.48
Vital parameters on admission, median (IQR)				
Respiratory rate	23 (20–30)	22 (20–34)	26 (24–32)	0.46
Heart rate	99 (90–117)	97 (81–109)	95 (87–114)	0.78
Mean arterial pressure	95 (86–123)	97 (91–106)	101 (90–130)	0.55

AHRF, acute hypercapnic respiratory failure; APACHE II, acute physiology and chronic health evaluation II; IQR, interquartile range 25th–75th percentile; NIV, noninvasive ventilation; SOFA, sequential organ failure assessment.

**Table 3:** Hospital length of stay and NIV duration, NIV parameters, and presence of delirium.

	Completed Group (*N*=20)	Chronic NIV Group (*N*=20)	Failed Group (*N*=11)	*P* value
Hospital length of stay and NIV duration, median (IQR)				
Hospital length of stay, days	10 (8–14)	11 (10–14)	11 (7–15)	0.76
NIV duration, days	4 (3–6)	11 (10–14)	2 (1–3)	**<0.01**
Number of days with 3 NIV sessions	1 (1-2)	1 (0–3)	1 (0–3)	0.96
Number of days with 2 NIV sessions	1 (1-2)	2 (1–9)	0 (0-1)	**<0.01**
Number of days with 1 NIV session	1 (1-2)	4 (0–5)	0 (0-1)	**< 0.01**
NIV parameters, median (IQR)				
Maximum IPAP (cm H_2_O)	16 (14–19)	21 (18–23)	17 (15–19)	**<0.01**
Maximum EPAP (cm H_2_O)	6 (5–7)	8 (7-8)	6 (5–7)	**<0.01**
IPAP (cm H_2_O) with domiciliary NIV	—	19 (17–23)	—	—
EPAP (cm H_2_O) with domiciliary NIV	—	8 (5–8)	—	—

EPAP, expiratory positive airway pressure; IQR, interquartile range 25th–75th percentile; IPAP, inspiratory positive airway pressure; NIV, noninvasive ventilation.

**Table 4:** Study outcomes.

Outcome, *n* (%)	Completed Group (*N*=20)	Chronic NIV Group (*N*=20)	Failed Group (*N*=11)	*P* value
Primary				
AHRF relapse after NIV discontinuation	0	0	3 (27)	**0.01**
In-hospital mortality	0	0	2 (18)	**0.03**
Secondary				
Readmission rate at 3 months	6 (30)	11 (55)	2 (22)	0.91
Mortality at 3 months	4 (20)	4 (20)	4 (44)	0.23

IQR, interquartile range 25th–75th percentile; AHRF, acute hypercapnic respiratory failure; NIV, noninvasive ventilation.

## Data Availability

The data used to support the findings of this study are available from the corresponding author upon request.

## Abbreviations

ABG: Arterial blood gases
AE-COPD: Acute exacerbation of chronic obstructive pulmonary disease
AHRF: Acute hypercapnic respiratory failure
APACHE II: Acute physiology and chronic health disease classification system II
CBM: Chart-based method
COPD: Chronic obstructive pulmonary disease
DNR/DNI: Do-not-resuscitate/do-not-intubate
EPAP: Expiratory positive airway pressure
FEV1: Forced expiratory volume in the 1st second
FiO_2_: Fraction of inspired oxygen
ICU: Intensive care unit
IPAP: Inspiratory positive airway pressure
IRCU: Intermediate respiratory care unit
IS: Incentive spirometry
NIV: Noninvasive ventilation
PaCO_2_: Arterial carbon dioxide partial pressure
PaO_2_/FiO_2_: Arterial oxygen partial pressure to fraction of inspired oxygen ratio
PEP: Positive expiratory pressure
RCT: Randomized controlled trial
RR: Respiratory rate
SpO_2_: Peripheral oxygen saturation
SOFA: Sequential organ failure assessment.

## References

[1] Davidson A. C., Banham S., Elliott M. (2016). BTS/ICS guideline for the ventilatory management of acute hypercapnic respiratory failure in adults. *Thorax*.

[2] Rochwerg B., Brochard L., Elliott M. W. (2017). Official ERS/ATS clinical practice guidelines: noninvasive ventilation for acute respiratory failure. *European Respiratory Journal*.

[3] Duan J., Tang X., Huang S., Jia J., Guo S. (2012). Protocol-directed versus physician-directed weaning from noninvasive ventilation. *Journal of Trauma and Acute Care Surgery*.

[4] Damas C., Andrade C., Araújo J. P., Almeida J., Bettencourt P. (2008). Desmame de ventilação não invasiva: experiência com períodos de descontinuação. *Revista Portuguesa de Pneumologia*.

[5] Sellares J., Ferrer M., Anton A. (2017). Discontinuing noninvasive ventilation in severe chronic obstructive pulmonary disease exacerbations: a randomised controlled trial. *European Respiratory Journal*.

[6] Lun C.-T., Chan V. L., Leung W.-S. (2013). A pilot randomized study comparing two methods of non-invasive ventilation withdrawal after acute respiratory failure in chronic obstructive pulmonary disease. *Respirology*.

[7] James J. (2016). Comprehensive geriatric assessment during emergency admission. *Nursing Older People*.

[8] Charlson M. E., Pompei P., Ales K. L., MacKenzie C. R. (1987). A new method of classifying prognostic comorbidity in longitudinal studies: development and validation. *Journal of Chronic Diseases*.

[9] Hurst J. R., Elborn J. S., Soyza A. D. (2015). COPD-bronchiectasis overlap syndrome. *European Respiratory Journal*.

[10] Segreti A., Stirpe E., Rogliani P., Cazzola M. (2014). Defining phenotypes in COPD: an aid to personalized healthcare. *Molecular Diagnosis & Therapy*.

[11] Knaus W. A., Draper E. A., Wagner D. P., Zimmerman J. E. (1985). Apache II: a severity of disease classification system. *Critical Care Medicine*.

[12] Vincent J.-L., Moreno R., Takala J. (1996). The SOFA (Sepsis-related Organ Failure Assessment) score to describe organ dysfunction/failure. *Intensive Care Medicine*.

[13] Global Initiative for Chronic Obstructive Lung Disease (2018). *GOLD 2017 Global Strategy for the Diagnosis, Management and Prevention of COPD*.

[14] Epstein L. J., Kristo D., Strollo P. J. (2009). Clinical guideline for the evaluation, management and long-term care of obstructive sleep apnea in adults. *Journal of Clinical Sleep Medicine*.

[15] Chanda A., Kwon J. S., Wolff A. J., Manthous C. A. (2012). Positive pressure for obesity hypoventilation syndrome. *Pulmonary Medicine*.

[16] Kelly B. J., Matthay M. A. (1993). Prevalence and severity of neurologic dysfunction in critically III patients. *Chest*.

[17] Morandi A., Solberg L. M., Habermann R. (2009). Documentation and management of words associated with delirium among elderly patients in postacute care: a pilot investigation. *Journal of the American Medical Directors Association*.

[18] Inouye S. K., Leo-Summers L., Zhang Y., Bogardus S. T., Leslie D. L., Agostini J. V. (2005). A chart-based method for identification of delirium: validation compared with interviewer ratings using the confusion assessment method. *Journal of the American Geriatrics Society*.

[19] Boles J.-M., Bion J., Connors A. (2007). Weaning from mechanical ventilation. *European Respiratory Journal*.

[20] Charlesworth M., Elliott M. W., Holmes J. D. (2012). Noninvasive positive pressure ventilation for acute respiratory failure in delirious patients: understudied, underreported, or underappreciated? A systematic review and meta-analysis. *Lung*.

